# Predictors of social response to COVID-19 among health care workers caring for individuals with confirmed COVID-19 in Jordan

**DOI:** 10.12688/f1000research.75740.1

**Published:** 2022-03-14

**Authors:** Heyam Dalky, Adam Khraisat, Anas H. Khalifeh, Sawsan Abuhammad, Ayman Hamdan-Mansour

**Affiliations:** 1 Community and Mental Health Department/Faculty of Nursing, Jordan University of Science and Technology, Irbid, 22110, Jordan; 2 Nursing Program/Health Science Faculty, Higher Colleges of Technology, Sharjah, United Arab Emirates; 3 Faculty of Nursing, Ministry of Health, Zarqa, Jordan; 4 Child Maternal Health Department, Jordan University of Science and Technology, Irbid, Jordan; 5 School of Nursing, The University of Jordan, Amman, Jordan

**Keywords:** Social Response, COVID-19, Health Care Workers, Coping, Jordan.

## Abstract

**Background: **The outbreak of COVID-19 forced public health authorities around the world to call for national emergency plans. Public responses, in the form of social discrimination and stigmatizing behaviors, are increasingly being observed against individuals with confirmed COVID-19 and healthcare workers (HCWs) caring for those individuals. Therefore, this study aimed to investigate the perception of social discrimination and coping strategies, and explore predictors of social discrimination and coping with COVID-19 among HCWs and individuals with confirmed COVID-19.

**Methods: **This study used a cross-sectional descriptive-comparative design to collect data, using a convenience sample of 105 individuals with confirmed COVID-19 and 109 HCWs using a web-based survey format.

**Results: **In this study, individuals confirmed with COVID-19 reported a high level of social discrimination compared with HCWs (t = 2.62, *p* < 0.01), while HCWs reported a high level of coping with COVID-19 compared to individuals with COVID-19 (t = -3.91, *p* < 0.001). Educational level, age, monthly income, and taking over-the-counter medication were predictors of social discrimination and coping with COVID-19 among HCWs and individuals with confirmed COVID-19.

**Conclusions:** The findings showed that individuals with confirmed COVID-19 were more likely to face social discrimination, and HCWs cope with COVID-19 better than ordinary individuals with confirmed COVID-19.

## Introduction

Over the past two years, the COVID-19 pandemic has been threatening human lives around the world, and health-care professionals are among those who are at higher risk due to immediate exposure to infection and infected people ( Gan *et al.*, 2020). Waves of COVID-19 outbreak are continuingly taking different forms and series adding further long-term burden on healthcare professionals. A recent study showed that the general population, due to COVID-19, were suffering stress, anxiety, depression, and psychological disturbances ( Hamaideh *et al.*, 2021). Nevertheless, health-care workers (HCWs) have demonstrated exceptional courage and commitment despite their fears of being at higher risk of infection and assumed to infect others ( Liu *et al.*, 2020). Approximately 14% of WHO-reported COVID-19 cases are HCWs ( Organization, 2020a). In addition, HCWs are also considered a source of virus transmission according to public perception ( Dalky *et al.*, 2020).

The increasing reports of spread of COVID-19 has provoked fear and concern among people globally ( Tang *et al.*, 2020). Important concerns include a stigma associated with COVID-19 and the issues related to unknown pathophysiology, treatment, and effectiveness of vaccination ( Dalky *et al.*, 2020; Organization, 2020b). This situation has created a new form of stigma and discrimination against individuals with COVID-19 ( Tang *et al.*, 2020). There has been a harsh attitude toward those who have been infected and HCWs caring for individuals confirmed with COVID-19 ( Bhanot *et al.*, 2020). The stigmatizing behaviors demonstrated the dread of the unknown and the unexplained negative attitudes toward infected people or suspected of being infected, as well as, those suspected of spreading the virus such as HCWs ( Bhanot *et al.*, 2020). Societies have blamed people infected with COVID-19 for being ignorant and indifferent ( Morin, 2021). This had indicated the need for more emphasis on research to understand the public responses and forms of understanding such dilemmas.

The pandemic has put an extraordinary psychological burden on HCWs due to work in high-demanding environments forcing them to isolate themselves for fear of transmitting the infection and further accusations of spreading the infection ( Rodríguez and Sánchez, 2020). In addition, it has been noticed that the use of social media to negatively reporting and addressing the COVID-19 pandemic has led to social discrimination against those confirmed with COVID-19 and HCWs, as well ( Singh and Subedi, 2020). Regrettably, HCWs are labeled, faced loss of status and discrimination due to the stigma associated with COVID-19 ( Singh and Subedi, 2020). Public responses in the form of social discrimination and stigmatizing behaviors are increasingly being observed against individuals with confirmed COVID-19 and HCWs caring for individuals with COVID-19 ( Abuhammad *et al.*, 2021; Bhanot *et al.*, 2020). Discriminatory behaviors are observed in the form of denial of infection, concealment of being infected, or refusal of the COVID-19 test. Thus, the need to emphasize such an issue would enable understanding reasons and contributing factors related to burnout and psychological distress among HCWs.

The ongoing transmission, the increasing number of COVID-19 cases, and the growing need to study the effects of community response, discrimination, and stigmatization against HCWs and individuals with confirmed COVID-19 infections are important areas that needs further exploration ( Dalky *et al.*, 2020). This makes addressing social discrimination practiced against individuals with COVID-19 and HCWs a significant issue ( Dalky *et al.*, 2020). Because of their long and intense exposure to various stressors, it is important to address and understand coping strategies used by HCWs and individuals with COVID-19 to manage stigma practiced against them. This will provide information guidance on what interventions could be implemented to maintain the best care for individuals infected with COVID-19 and maintaining the mental wellness of HCWs, as well. Although studies are increasingly published in the context of psychological consequences of COVID-19 worldwide, there is still a need to address public responses and how it is affecting HCWs’ coping abilities; in particular, in the Arab culture. This study investigated the perception of social discrimination and coping strategies, and predictors of social discrimination and coping toward COVID-19 among HCWs and individuals with COVID-19.

The aims of this study were:

• To compare coping and social discrimination to COVID-19 among HCWs and individuals with COVID-19.
• To assess sociodemographic or personal factors that could predict coping and social discrimination toward COVID-19 among HCWs and individuals with COVID-19.

## Methods

### Design

This study utilized a cross-sectional design using a descriptive-comparative approach of research. Data collected in relation to social discrimination and coping from individuals infected with COVID-19 and their health-care workers caring form them was collected using a self-reported online survey format.

### Sample and setting

The sample included individuals who had been infected with COVID-19 and HCWs caring of individuals with COVID-19. A convenience sampling technique was used to recruit the participants of this study. Inclusion criteria for HCWs were: 1) providing direct care to those infected with COVID-19. HCW were excluded if he/she reported being infected. For public, to be eligible, required 1) being aged 18 years or above, 2) have access to software to fill out the survey, and 3) be able to read and write in Arabic. Those with physical, mental or cognitive disabilities were excluded as it may interfere with their ability to understand the questions and make their responses.

### Instruments

The Arabic versions of the tools were used in this study. WHO guidelines for translation and adaption were used to translate the survey. The tools used were as follows:

Social discrimination was measured using the Social Discrimination Scale ( Dinos *et al.*, 2004). The Arabic version of the social discrimination subscale was developed and used before by Dalky et al. (2020) and thus used in this study. This subscale is composed of 12 items and individuals are requested to choose their response on a Likert scale formed of five possible responses ranging from 1 (strongly disagree) to 5 (strongly agree). Those who had a higher score on the scale were more likely to experience a higher level of social discrimination. Previous studies showed good internal consistency with Cronbach's alpha of 0.80 ( Dalky *et al.*, 2020).

Lifestyle management and psychosocial adaptation with COVID-19 were measured using the FANTASTIC survey ( Wilson *et al.*, 1984). This scale focuses on physical and health-related fitness of individuals. The scale is formed of 17 items measuring nine domains with each domain name represented by the first letter of the word “FANTASTIC”: F for Family and Friends, A for Activity, N for Nutrition, T for Tobacco and Toxics, A for Alcohol Intake, S for Sleep, Seatbelts, Stress, and Safe sex, T for Type of behavior, I for Insight, and C for Career. The scale investigates individuals' perception during the previous month. The total score indicates the category in which the individual falls, ranging from needing improvement (0-35) to excellent (85-100). The scale has good reliability with Cronbach's alpha of *r* = 0.88.

*Sociodemographic variables*

An author-developed profile was developed to collect information in relation to age, gender, marital status, and other sociodemographic information from both patients and HCWs. Specific information was collected from HCWs regarding their work placement and experiences.

### Data analysis

The software package IBM-SPSS v.25 was used to analyze the collected data. Central tendency measures and dispersion measures were used to describe the variables of the study. Pearson‘s *r* coefficient was used to assess correlation magnitude and direction. A t-test for two independent samples and ANOVA were used to test differences and compare the HCWs’ and individuals with COVID-19 responses, respectively. A multiple logistic regression test was used to examine predictors of social discrimination. The alpha significance level was set to 0.05.

The study was granted ethical approval (reference number 113/132/2020), as suggested by both academic authors’ institutions and hospitals administration systems relevantly. The authors received written informed consent from HCWs and individuals with confirmed COVID-19 participants. The consent form included detailed information about the study aims, and the need to conduct such studies, as well as their approval for the publication of this manuscript for research purposes to increase knowledge in this area of COVID-19 impact.

## Results

### Demographic variables

In this study, the total number of individuals with confirmed COVID-19 who completed the survey was 105. Their age ranged from 18-60 years with a mean (M) of 34 (SD=10.4) years (see Table 1). The total number of HCWs who participated was 109. The age of HCWs ranged from 23-65 years with a mean of 33.7 (SD = 7.6) years (see Table 2).

**Table 1.** Descriptive statistics of Individuals with confirmed COVID-19 (N=105). JD = Jordan Dinar.

	*n*	*%*
***Gender***		
Female	49	46
Male	56	54
***Education level***		
High school	15	14.3
Diploma	16	15.2
Bachelor	61	58.1
Graduate studies	13	12.4
***Marital status***		
Single	32	30.5
Married	65	61.9
Divorced	4	3.8
Widowed	4	3.8
***Monthly income***		
Less than 200 JD	21	20.0
From 201 to 500	53	50.5
From 501 to 800	26	24.8
From 801 to 1200	5	4.8
More than 1201	21	20.0
***Quarantine***		
Not quarantined-not infected	69	65.7
Quarantined-not infected	36	34.3
Quarantined for screening	6	2.2

**Table 2.** Health-care workers descriptive statistics (N=109).

	*n*	*%*
***Gender***		
Male	46	42.2
Female	63	57.8
***Marital status***		
Single	27	24.8
Married	77	70.6
Divorced	5	4.6
***Working area***		
Governmental hospital	44	40.4
Military hospital	19	17.4
Private sector	25	22.9
University-affiliated	18	16.5
***Education level***		
Diploma	13	11.9
Bachelor	76	69.7
Graduate	20	18.3
***Specialty***		
Nurse	91	83.5
Physician	7	6.4
Radiology technician	2	1.8
Midwife	4	3.7
Pharmacist	2	1.8
Administrator	3	2.8

### Differences between HCWs and individuals with confirmed COVID-19 in relation to social discrimination associated with COVID-19

The results of the t-test showed that there was a significant difference between mean score of HCWs and individuals with confirmed COVID-19 in relation to social discrimination (t = 2.62, p < 0.01) (see Table 3). The total mean item score of individuals with confirmed COVID-19 was higher (M = 2.64, SD = 0.867) than the total mean item score of HCWs (M = 2.07, SD = 0.19). The item-to-item comparison showed that individuals with confirmed COVID-19 mean item scores were significantly higher than HCWs in all items. The highest mean scores for individuals with confirmed COVID-19 were observed for the items “People insulted me for being diagnosed with the Coronavirus” and “I have not had any problems due to Coronavirus diagnosis” (M = 3.87, SD = 0.856; M = 3.66, SD = 1.192; respectively). The lowest mean items for individuals with confirmed COVID-19 were observed for “I was discriminated against because of my diagnosis of the Coronavirus” and “After I suffered due to a diagnosis of Coronavirus, I feel that life is unfair” (M = 1.99, SD = .098; M = 2.74, SD = 1.010; respectively).

**Table 3.** Differences between health-care workers (HCWs) (N=109) and individuals with confirmed COVID-19 (N = 105) in perception of social discrimination for people with COVID-19.

Items	HCW		Individuals with COVID-19		t-test	*p*-value
	M	SD	M	SD		
1. I was discriminated against because of my diagnosis of the Corona virus	1.96	1.20	1.99	.098	2.62	< .01
2. Sometimes I feel uncomfortable talking to them because of my diagnosis of the Coronavirus	2.30	1.18	3.51	1.084		
3. I was discriminated against by the police because of my diagnosis of Corona virus	1.99	1.14	3.34	1.167		
4. I was discriminated against by my employer because of my diagnosis of Coronavirus	1.93	1.05	2.99	1.213		
5. I often feel lonely because of my diagnosis of Corona virus	2.14	1.19	3.22	1.208		
6. People’s reactions to being diagnosed with the Coronavirus make me keep myself isolated	2.37	1.13	3.34	.979		
7. I am angry at the way people interacted with me for being diagnosed with the Coronavirus	2.11	1.09	3.24	1.079		
8. I have not had any problems due to Corona virus diagnosis	2.17	1.18	3.66	1.192		
9. I have been discriminated against by health professionals due to my diagnosis of Coronavirus	1.86	1.05	3.07	1.040		
10. People have avoided me because I am diagnosed with the Coronavirus	2.26	1.13	3.16	1.234		
11. People insulted me for being diagnosed with the Coronavirus	1.75	.87	3.87	.856		
12. After I suffered due to a diagnosis of Coronavirus, I feel that life is unfair	1.97	1.13	2.74	1.010		
***Social discrimination total***	2.07	.19	2.64	.867		

### Differences between HCWs and individuals with COVID-19 in relation to coping strategies

The results of the t-test showed that there was a significant difference between HCWs and individuals with confirmed COVID-19 in coping with COVID-19 (t = -3.91, *p* < .001) (see Table 4). The total mean item score of HCWs was higher (M= 51.8, SD = 0.86) than the total mean coping item score of individuals with confirmed COVID-19 (M = 34.3, SD = 1.19). The item-to-item comparison showed that, in general, individuals with COVID-19 had significantly lower mean item scores than HCWs for all items. The highest mean items for HCWs were observed for “I am satisfied with my job or role” and “I give and receive affection” (M = 4.28, SD = 0.859; M = 4.14, SD = 0.976), respectively. The lowest mean item scores for HCWs were observed for “I am vigorously active for at least 30 minutes per day e.g., running, cycling, etc.” and “I eat a balanced diet (see explanation)” (M = 1.56, SD = 1.013; M = 2.44, SD = 1.287), respectively.

**Table 4.** Differences between health-care workers (HCWs) and indiveduals with confirmed COVID-19 in relation to coping with COVID-19, (N= 214).

Items	Infected people		HCW		t-test	*p-*value
	M	SD	M	SD		
1. I have someone to talk to about things that are important to me	3.69	1.022	4.10	1.071		
2. I give and receive affection	3.78	.940	4.14	.976		
3. I am vigorously active for at least 30 minutes per day e.g., running, cycling, etc	1.65	1.015	1.56	1.013		
4. I am moderately active (gardening, climbing stairs, walking, housework)	2.01	1.312	2.80	1.556	t = -3.91	*p* < .001
5. I eat a balanced diet (see explanation)	2.06	1.110	2.44	1.287		
6. I often eat excess 1) sugar, or 2) salt, or 3) animal fats, or 4) junk food	2.33	1.141	2.64	1.183		
7. I sleep well and feel rested	3.33	.927	3.27	1.168		
8. I use seatbelts	3.34	1.377	3.01	1.309		
9. I can cope with the stresses in my life	3.48	.856	3.89	.906		
10. I relax and enjoy leisure time	3.12	.927	3.17	1.096		
11. I practice safe sex (see explanation)	2.91	1.201	3.21	1.667		
12. I seem to be in a hurry	2.93	.858	2.75	1.073		
13. I feel angry or hostile (Reversed)	3.18	.838	3.28	.983		
14. I am a positive or optimistic thinker	3.39	.766	3.84	.973		
15. I feel tense or uptight (Reversed)	2.96	.950	2.94	.993		
16. I feel sad or depressed (Reversed)	3.05	.974	3.11	1.117		
17. I am satisfied with my job or role	3.51	.942	4.28	.859		

### Predictors of coping with COVID-19 among HCWs and individuals with confirmed COVID-19

Regarding predictors of coping with COVID-19 in relation to demographic variables of the whole group, a multiple regression test was conducted. The results showed that the model was significant (F = 14.88, *p* = 0.001). Educational level was a significant predictor (B = 0.541, *p* < 0.001) indicating that those with higher educational levels had higher scores in coping with COVID-19. Age was also a significant predictor (B = 0.187, *p* =0.007) indicating that older people have higher coping score. The third significant predictor was monthly income (B= -0.338, *p* < 0.001) that has negative association imposing income as risk factor. In addition, taking over-counter medication was a significant predictor (B = 0.54, *p* < 0.001) indicating that those who use over-counter medication are more likely to have higher coping scores (see Table 5).

**Table 5.** Predictors of coping among health-care workers (HCWs) and individuals with confirmed COVID-19 (n= 214).

Model		Unstandardized coefficients		Standardized coefficients	t	Sig.
		B	standard error	Beta		
1	(Constant)	29.351	10.951		2.680	.008 *
	Age	.179	.066	.187	2.712	.007
	Gender	.862	1.353	.039	.637	.525
	Marital status	.098	1.270	.005	.077	.939
	Monthly income	-4.109	.677	-.338	-6.070	.000 **
	Medication over counter	4.865	.506	.541	9.614	.000 **

Dependent variable: SUMCOPING.

* *p <* 0.01.

** *p* < 0.001.

### Predictors of discrimination perception associated with COVID-19 among HCWs and individuals with confirmed COVID-19

To investigate discrimination perception associated with COVID-19 among both groups, a multiple regression test was conducted. The results showed that the model was significant (F =14.21, *p* = .001). The predictors were educational level (B = -0.447, *p* < 0.001) which indicated that as the level of education increased, individuals experienced less discrimination associated with COVID-19; age (B = -0.162, *p* = 0.02): as age increased the discrimination against COVID-19 was decreased; and monthly income (B = 452, *p* < 0.001), which means with a higher income experienced more discrimination associated with COVID-19. The other predictor was taking medication over the counter (B = 0.447, *p* < 0.001) which means people taking medication over the counter experienced more discrimination associated with COVID-19 (see Table 6).

**Table 6.** Predictors of discrimination against COVID-19 among health-care workers (HCWs) and individuals with confirmed COVID-19 (N= 214).

Model		Unstandardized coefficients		Standardized coefficients	t	Significant level
		B	Std. Error	Beta		
1	(Constant)	44.555	11.656		3.823	.000 **
	Age	-.164	.070	-.162	-2.334	.021
	Gender	-1.115	1.440	-.048	-.774	.440
	Marital status	1.737	1.351	.090	1.285	.200
	Monthly income	5.792	.721	.452	8.039	.000 **
	Medication over counter	4.237	.539	.447	7.866	.000 **

Dependent variable: SUMD.

** *p* < 0.001.

## Discussion

Responses to COVID-19 might influence the process of treatment and willingness to collaborate. Therefore, differences in perception of social responses in the form of social discrimination between HCWs and individuals with confirmed COVID-19 is a core component in coping with the disease. The results of this study showed that individuals with confirmed COVID-19 had a higher perception of social discrimination and a lower level of coping with COVID-19 compared to HCWs. The results indicate that the general and ordinary individuals are more likely to be exposed to discrimination and possess a lower level of ability to cope with the disease and related factors than the HCWs. Moreover, it is possible that HCWs might have a higher level of knowledge and competency to manage discrimination phrases or cues or have lower sensitivity for such expressions that contributed to their feelings of being discriminated against. It is expected due to their education and training that HCWs are more capable of coping with the disease and manage discrimination than the general population ( Chew *et al.*, 2020). Such major differences in perception of being discriminated against may cause conflicts in understanding, communicating, or commitment to a treatment plan that interferes with the achievement of health-care outcomes. The results do support previous reports in which social discrimination and fear of communicable diseases hampered the response of the public ( Brooks *et al.*, 2020; Liebrenz *et al.*, 2020). It has also been noted that social discrimination has forced people to negate their positive results of infection to avoid discrimination leading them to avoid seeking healthcare services and lacking protective health measures that endangered others' health conditions and lives ( Brooks *et al.*, 2020; Liebrenz *et al.*, 2020).

While COVID-19, due to its pandemic nature and global influence pattern, is considered a stress-inducing illness, coping strategies are still required to manage the disease and its consequences ( Hamaideh et al., 2021). In this study, HCWs used coping strategies more effectively than individuals with COVID-19. In previous reports, HCWs were challenged in managing their responsibilities due to stigma and discrimination ( Dalky *et al.*, 2020). Nevertheless, they were able to better use the coping strategies than ordinary people. It is worth saying that social isolation and job burden are factors that have contributed to increased job stress among HCWs ( Bani-Hani and Hamdan-Mansour, 2021; Singh and Subedi, 2020). Thus, HCWs might have depended largely on their learned adaptation skills to manage job stress to be able to handle discrimination and cope better COVID-19. On the other hand, ordinary people probably lack the skills and knowledge to manage discrimination due to fear of losing their social and occupational privileges ( Chew *et al.*, 2020). There were differences in using coping strategies between HCWs and individuals with confirmed COVID-19. Such difference might negatively affect HCWs, due to the fact that they are more vulnerable to COVID-19 infection compared to the general population. It has been confirmed that such differences might influence and explain the level of care provided by the HCWs and the low level of compliance and collaboration of individuals with confirmed COVID-19 ( Kar *et al.*, 2021). The degree to which a person fears COVID-19 is an element that could be important in understanding the coping process. Fear is an emotional state that stimulates self-defense behaviors; thus, fear of infection would influence coping strategies leading to poor prognosis ( Kim *et al.*, 2020).

Besides, many factors could be related to social discrimination against individuals with confirmed COVID-19 or HCWs caring for those people. Such factors might be related to the collectivist culture that puts high social pressure on people and affects their decisions to seek healthcare services ( Al Ali *et al.*, 2017; Aldalaykeh *et al.*, 2019). For instance, fear and anxiety from the spread of COVID-19 may lead to social discrimination in people having disease, places that are considered sources of the infection such as hospitals, and even people who were in quarantine ( CDC, 2020). Furthermore, social discrimination and stigmatized behaviors are extensively noted in mental health research; however, these variables in the context of COVID-19 seem to lead to the same negative effect on health-care outcomes ( Brooks *et al.*, 2020). The notion that social discrimination is a multifaceted factor infers that a reciprocal relationship exists between the bio-psycho-social and cultural components of human wellbeing ( Dalky *et al.*, 2020). In other words, the social discrimination might take various forms depending on the cultural definition of discrimination. People with COVID-19 who complied with the quarantine have reported higher levels of psychological disturbances such as stress and anger ( Brooks *et al.*, 2020). Such a critical health situation in addition to poor coping and discrimination, might be a threat to successful endorsement and implementation of public healthcare plans against COVID-19 leading to poor healthcare outcomes ( Fu *et al.*, 2021).

Furthermore, the study found that HCWs and individuals with confirmed COVID-19 who have a high level of education and use over-the-counter medication showed greater coping with COVID-19. The results support previous equivalent studies that indicated using emotional-based coping among low educated people compared to using problem-based coping among those with a high level of education ( Mohammadzadeh *et al.*, 2020; Shamsi *et al.*, 2021). This reflects the differences in the behavioral responses related to individual knowledge and experience. In addition, the results showed that HCWs who earned a high income showed greater coping with COVID-19. These findings are in line with previous studies that found financial constraints were linked to and predicted higher levels of stress and lower levels of effective coping ( Atchison *et al.*, 2020; Barbara *et al.*, 2020; Cluver *et al.*, 2020). Similarly, age and literacy play a positive role in predicting effective coping and positive responses ( Darraj *et al.*, 2016; Magsamen-Conrad *et al.*, 2019; Moukaddam and Shah, 2020; Volk *et al.*, 2021), older people and more literate ones were found to use more effective coping strategies with COVID-19 than younger and illiterate people. Such findings were consistent with previous studies that reported a higher level of coping connected to higher levels of resiliency ( Pearman *et al.*, 2020).

### Limitations

There were two limitations of our study. First, data was collected using cross sectional sampling utilizing an online survey format which limits ability to derive causal relationships. Secondly, using a self-reporting format might not allowed to draw objective data affected by recall bias.

## Conclusions

This study focused on exploring predictors of coping and responses to social discrimination in the form of stigma among HCWs and individuals with confirmed COVID-19. The findings showed that individuals with COVID-19 were more likely to face social discrimination than HCWs. Yet, in dealing with COVID-19, HCWs used more effective coping strategies with COVID-19 than non-medical infected individuals. The main conclusion of this study is that predictors of social discrimination and coping were educational level, age, monthly income, and taking over-the-counter medication. Although both social discrimination and coping are complicated and may be influenced by a variety of factors, we must reconsider and find ways to reinforce them in light of the probable recurrence of COVID-19 and other future global pandemic risks. Innovative strategies are to be granted to clinical practice and the public sectors to best tackle the challenges associated with the COVID-19 pandemic. In addition, public media also has to be targeted to combat discrimination and support individuals confirmed with COVID-19 and HCWs caring for them.

### Relevance for clinical practice

This study has implications relevant to clinical practice, as well as policymakers and public health officers. The results emphasize the need to enhance mutual understanding of the effect of discrimination on both ordinary people and HCWs. Training and enhancement of psychological skills is needed for both ordinary people and HCWs and has to be included in treatment protocols. Furthermore, HCWs are in need of peer and organization support to enable them to manage job burden and discrimination. Management and administrative personnel can guide and support the HCWs by reforming or modifying the current clinical practice to best accommodate and cope with extra or unexpected demands added to the HCWs shoulders, as those seen during the COVID-19 pandemic.

## Data availability

### Underlying data

Zenodo: Data repository of Predictors of Social Response to COVID-19 among Health Care Workers Caring for Individuals with Confirmed COVID-19 in Jordan, https://doi.org/10.5281/zenodo.6044084 ( Dalky *et al.*, 2022).

This project contains the following underlying data:

- Project1 (2) (1).xlsx (raw survey data)

### Extended data

Zenodo: Data repository of Predictors of Social Response to COVID-19 among Health Care Workers Caring for Individuals with Confirmed COVID-19 in Jordan, https://doi.org/10.5281/zenodo.6044084 ( Dalky *et al.*, 2022)

This project contains the following extended data:

- COVID-19 in Jordan.docx

Data are available under the terms of the Creative Commons Attribution 4.0 International license (CC-BY 4.0).
