# Supplementary material for: Predictors of social response to COVID-19 among health care workers caring for individuals with confirmed COVID-19 in Jordan
Source: F1000Res. 2022 Apr 25;11:312. Originally published 2022 Mar 14. [Version 2] doi: 10.12688/f1000research.75740.2 (PMC10015117; doi:10.12688/f1000research.75740.2)
Supplement: Peer review history

# Peer review: Reviewer response for version 2

**DOI:** 10.5256/f1000research.133419.r161583

**Published:** 2023-03-14

**Authors:** Richard Mottershead

**Affiliations:** 1 Ras Al Khaimah College of Nursing, RAK Medical and Health Sciences University, Ras Al Khaimah, United Arab Emirates

Thank you for allowing me the opportunity to review this research article paper which I have found both insightful and informative. I have read the article with interest and have the following comments to deliver:

 I believe that this is a well-composed article, which will be of interest to all healthcare practitioners and the general populations as we attempt to gain an understanding on the impact and fallout of covid-19. The authors are embedded within clinical practice and education and seek to make long-standing contribution to this crucially important field of research that is both contemporary and adds new knowledge to the evidence based literature. This of course will aid and inform clinical practice if assimilated by health and social care professionals and policy decision makers. The paper provides insight into the lived experience of HCW's and social discrimination within Jordan and allows for comparative overview with studies conducted outside the MENA region. The paper supports a collective consciousness on the impact of the pandemic on our health and social care workers and allows for consideration to be given for how they can be supported in future pandemics.

 The authors identify the limitations of the study and it is feasible that future studies make seek a narrative/life story approach to gain further mean-making insight into the HCW's lives. The research could assist with resilience and coping strategies to improve service delivery by combating stigmatisation.  

 I hope that the paper allows the authors to continue their research journey and I wish the authors well.

Is the work clearly and accurately presented and does it cite the current literature?

Yes

If applicable, is the statistical analysis and its interpretation appropriate?

Yes

Are all the source data underlying the results available to ensure full reproducibility?

No source data required

Is the study design appropriate and is the work technically sound?

Yes

Are the conclusions drawn adequately supported by the results?

Yes

Are sufficient details of methods and analysis provided to allow replication by others?

Yes

Reviewer Expertise:

I am a qualified researcher, health practitioner with experience of supporting health and social care practitioners during covid 19 and published within this research topic.

I confirm that I have read this submission and believe that I have an appropriate level of expertise to confirm that it is of an acceptable scientific standard.

# Peer review: Reviewer response for version 1

**DOI:** 10.5256/f1000research.79654.r127956

**Published:** 2022-04-12

**Authors:** Sergul Duygulu

**Affiliations:** 1 Department of Nursing Management, Hacettepe University Faculty of Nursing, Ankara, Turkey

Thank you very much for giving me the opportunity to review this manuscript. The subject covered in the study is a current and important issue. In particular, the research has addressed an important issue in terms of addressing the problems experienced by healthcare professionals and individuals diagnosed with COVID-19 at the beginning of the pandemic.

 This study aimed to explore perceptions of social discrimination and coping strategies, and to explore predictors of social discrimination and coping with COVID-19 among healthcare workers and individuals with confirmed COVID-19. The theoretical basis of the study is well explained and it is clearly stated that the results of the study will meet which need in the literature. However, it can be put forward more clearly why the determinants of social discrimination and coping with COVID-19, which are discussed in this study, should be determined. Another suggestion is that in this study, there is a need to include the answers to the questions of why individuals and healthcare professionals with a history of being diagnosed with COVID-19 and healthcare professionals at the same time and why a comparative study is needed, albeit briefly, in the introduction.

 The aim of the study is stated. However, it would be appropriate to include research questions/hypotheses.

 The design of the study is appropriate. Sampling inclusion and exclusion criteria are specified. There is no statement that the adequacy of the sample size is evaluated. It would be appropriate to write. The study's sample “individuals with confirmed with COVID 19 and healthcare workers” is considered to be the limitation of this study. However, this limitation is acceptable as the results of the study address an important issue. However, as a limitation, it would be appropriate to refer to the sample size in the limitation session of manuscript.

 Although it has been stated that data collection is carried out online, the data collection procedure can be explained in more detail. How did the process work?

 The findings, discussion and conclusion of the study were reported in accordance with the aims of the study.

Is the work clearly and accurately presented and does it cite the current literature?

Yes

If applicable, is the statistical analysis and its interpretation appropriate?

Yes

Are all the source data underlying the results available to ensure full reproducibility?

No source data required

Is the study design appropriate and is the work technically sound?

Yes

Are the conclusions drawn adequately supported by the results?

Yes

Are sufficient details of methods and analysis provided to allow replication by others?

Partly

Reviewer Expertise:

Nursing management, community health nursing

I confirm that I have read this submission and believe that I have an appropriate level of expertise to confirm that it is of an acceptable scientific standard.

# Response

**Published:** 2022-04-21

**Authors:** Heyam Dalky

**Affiliations:** Jordan University of Science and Technology, Jordan

Thank you very much for giving me the opportunity to review this manuscript. The subject covered in the study is a current and important issue. In particular, the research has addressed an important issue in terms of addressing the problems experienced by healthcare professionals and individuals diagnosed with COVID-19 at the beginning of the pandemic.

****

 This study aimed to explore perceptions of social discrimination and coping strategies and to explore predictors of social discrimination and coping with COVID-19 among healthcare workers and individuals with confirmed COVID-19. The theoretical basis of the study is well explained and it is clearly stated that the results of the study will meet which needs in the literature. However, it can be put forward more clearly why the determinants of social discrimination and coping with COVID-19, which are discussed in this study, should be determined.

**Response**: Social discrimination has been reported widely in the literature during the pandemic and people reacted differently to the diagnosis. We thought HCP also might have this and decided to study it and also the predictors to better plan for future occurrences of the unexpected pandemics.

 Another suggestion is that in this study, there is a need to include the answers to the questions of why individuals and healthcare professionals with a history of being diagnosed with COVID-19 and healthcare professionals at the same time and why a comparative study is needed, albeit briefly, in the introduction.

**Response**: It is known in the literature that experience or perception of social discrimination and stigma are personal in nature and varies among individuals or groups. This perception could lead to various negative consequences that require further investigations to study and plan the suitable interventions to tackle and treat them. Yet, a comparative study or approach usually presents differences among both groups to highlight the interventions needed accordingly.

 The aim of the study is stated. However, it would be appropriate to include research questions/hypotheses.

**Response**: This comment is fully considered. The requested information is added in red-colored text after the study aims in the introduction section.

 Research Questions:

- What is the level of social discrimination against COVID-19 among HCWs and individuals with COVID-19?
- What is the level of coping with COVID-19 among HCWs and individuals with COVID-19?
- Which are the study factors that could predict coping and social discrimination toward COVID-19 among HCWs and individuals with COVID-19?

The design of the study is appropriate. The sampling inclusion and exclusion criteria are specified. There is no statement that the adequacy of the sample size is evaluated. It would be appropriate to write.

**Response**: This information is added as suggested. See red-colored text in the methods section; sample.

 A minimum number of 100 per each group was determined as necessary to achieve the study's desired power of 80 % at the level of significance of α =0.05.

 The study's sample of “individuals with confirmed with COVID 19 and healthcare workers” is considered to be the limitation of this study. However, this limitation is acceptable as the results of the study address an important issue. However, as a limitation, it would be appropriate to refer to the sample size in the limitation session of manuscript.

**Response**: These comments are fully considered. The sample size in this study was convenient and appropriate to drown the conclusions derived.

 Although it has been stated that data collection is carried out online, the data collection procedure can be explained in more detail. How did the process work?

**Response**: These comments are fully considered, the information is added for more clarifications. See methods added red-text.

 In this study, data was collected via an online survey. Participants were contacted online via social media available for HCWs and individuals with COVID-19. Upon agreeing to participate in the study, signed the consent participants were encouraged to complete the online survey. Authors' information was available shall they have questions or queries and assistance was available accordingly.

 The findings, discussion and conclusion of the study were reported in accordance with the aims of the study.

- Is the work clearly and accurately presented and does it cite the current literature?
  Yes
- Is the study design appropriate and is the work technically sound?
  Yes
- Are sufficient details of methods and analysis provided to allow replication by others?
  Partly
- If applicable, is the statistical analysis and its interpretation appropriate?
  Yes
- Are all the source data underlying the results available to ensure full reproducibility?
  No source data required
- Are the conclusions drawn adequately supported by the results?
  Yes
